# The LUNET Project: Developing the Italian Systemic Erythematous Lupus Network

**DOI:** 10.3390/jcm14176197

**Published:** 2025-09-02

**Authors:** Ilaria Mormile, Luisa Brussino, Giorgio Walter Canonica, Francesca Cortini, Maria Teresa Costantino, Lorenzo Dagna, Stefano Del Giacco, Francesca Della Casa, Mario Di Gioacchino, Giacomo Emmi, Gianluca Moroncini, Simone Negrini, Daniela Pacella, Paola Parronchi, Vincenzo Patella, Francesca Wanda Rossi, Concetta Sirena, Massimo Triggiani, Angelo Vacca, Amato de Paulis

**Affiliations:** 1 Department of Translational Medical Sciences, Federico II University, 80131 Naples, Italy; francescadellacasa4@gmail.com (F.D.C.); francescawrossi@gmail.com (F.W.R.); depaulis@unina.it (A.d.P.); 2 SCDU Immunologia e Allergologia AO Ordine Mauriziano, University of Turin, 10128 Turin, Italy; luisa.brussino@unito.it (L.B.); simone.negrini@unito.it (S.N.); 3 Dipartimento di Scienze Mediche, University of Turin, 10124 Turin, Italy; 4 Department of Biomedical Sciences, Humanitas University, 20072 Pieve Emanuele, Italy; giorgio_walter.canonica@hunimed.eu; 5 Personalized Medicine, Asthma and Allergy, Humanitas Clinical and Research Center IRCCS, 20089 Rozzano, Italy; 6 In&Fo&Med, Via San Gregorio 12, 20124 Milan, Italy; francesca.cortini@infomed-online.it (F.C.); concetta.sirena@infomed-online.it (C.S.); 7 Allergology, Clinical Immunology and Reumatology Unit, Carlo Poma Hospital, 46100 Mantua, Italy; mariateresa.costantino@asst-mantova.it; 8 Unit of Immunology, Rheumatology, Allergy and Rare Diseases (UnIRAR), IRCCS Ospedale San Raffaele, 20132 Milan, Italy; dagna.lorenzo@unisr.it; 9 Faculty of Medicine, Vita-Salute San Raffaele University, 20132 Milan, Italy; 10 Department of Medical Sciences and Public Health, University of Cagliari, 09124 Cagliari, Italy; delgiac@gmail.com; 11 Synergo-Institute of Clinical Immunotherapy and Advanced Biological Treatments, 66100 Pescara, Italy; digioacchino@me.com; 12 Department of Medical, Surgical and Health Sciences, University of Trieste, 34100 Trieste, Italy; 13 Clinical Medicine and Rheumatology Unit, Cattinara University Hospital, 34100 Trieste, Italy; 14 Centre for Inflammatory Diseases, Monash University Department of Medicine, Monash Medical Centre, Melbourne 3168, Australia; 15 Department of Clinical and Molecular Sciences, Marche Polytechnic University, 60121 Ancona, Italy; g.moroncini@univpm.it; 16 Department of Internal Medicine, Marche University Hospital, 60126 Ancona, Italy; 17 Department of Public Health, University of Naples Federico II, Via Pansini 5, 80131 Naples, Italy; 18 Department of Experimental and Clinical Medicine, University of Florence, 50134 Florence, Italy; paola.parronchi@unifi.it; 19 Immunology and Cell Therapy Unit, Careggi University Hospital, 50139 Florence, Italy; 20 Department of Internal Medicine ASL Salerno, ‘Santa Maria Della Speranza’ Hospital, 84091 Salerno, Italy; patella@allergiasalerno3.it; 21 Postgraduate Programme in Allergy and Clinical Immunology, University of Naples Federico II, 80131 Naples, Italy; 22 Center for Basic and Clinical Immunology Research (CISI), University of Naples Federico II, 80131 Naples, Italy; 23 WAO Center of Excellence, 80131 Naples, Italy; 24 Division of Allergy and Clinical Immunology, University of Salerno, 84084 Fisciano, Italy; mtriggiani@unisa.it; 25 Department of Precision and Regenerative Medicine and Ionian Area, UOC Medicina Interna “Guido Baccelli”, University of Bari Aldo Moro, Policlinico, 70126 Bari, Italy; angelo.vacca@uniba.it

**Keywords:** systemic erythematosus lupus, registry, network, biologics

## Abstract

Systemic lupus erythematosus (SLE) is a complex autoimmune disease that affects multiple organs and systems with a broad and heterogeneous spectrum of clinical manifestations. National disease-specific datasets and registries are crucial for clinical research since they can provide real-world and long-term data about clinical aspects, biomarkers, and treatments. Registries collect data from actual patients over time, outside the controlled environment of randomized controlled trials. This can help enhance the understanding of the natural history of a disease, provide information about how treatments work in everyday settings and elucidate potential variations in care and outcomes across different geographic areas. Here, we present a protocol for the creation of a standardized national disease-specific dataset for patients with SLE—the Systemic Lupus Erythematous Network (LUNET) Registry—which will facilitate data sharing, cross-comparison, and interoperability among centers. The LUNET registry is intended to serve as a comprehensive primary data source, capturing real-world longitudinal clinical information and the heterogeneity of patient presentations that are often underrepresented in traditional clinical trials. Ultimately, the LUNET registry will help to optimize SLE management in routine clinical practice by enabling the compilation of real-world evidence to inform clinical decision-making and health policy.

## 1. Introduction

Systemic lupus erythematosus (SLE) is a chronic autoimmune disease characterized by immune system dysregulation [1,2]. The presentation includes a broad and heterogeneous spectrum of clinical manifestations that can affect several organ systems and that pose substantial morbidity and mortality risk [3]. Preventing long-term organ dysfunction requires early diagnosis, along with prompt and targeted treatment. Due to the multisystemic nature of SLE, case management should entail a multidisciplinary approach that integrates expertise from different specialists [3]. To support early diagnosis and assessment of disease activity, recent studies have investigated several biomarkers that seem promising based on our improved understanding of the pathogenic mechanisms underlying SLE development [4,5]. Additionally, in recent years, the therapeutic armamentarium has substantially broadened. Notably, biological-targeted drugs (i.e., belimumab and anifrolumab), together with conventional disease-modifying antirheumatic drugs (cDMARDS), have enabled improved management of SLE patients, with treatment tailored according to their clinical phenotypes [5,6]. In this era of precision medicine, it is increasingly important to perform early patient characterization based on clinical and laboratory features, thereby enabling prediction of long-term prognosis and therapeutic outcomes [7].

The greater availability of effective and safe drugs has significantly increased patients’ survival; however, mortality remains higher among patients with SLE compared to in the general population, and new causes of death have emerged, which must be further explored to improve prevention strategies [8]. Moreover, certain clinical manifestations associated with specific organ involvement—e.g., neuropsychiatric systemic lupus erythematosus (NPSLE)—have not yet been fully characterized, due to the heterogeneity of definitions and study populations in the existing literature [9,10,11]. In the present paper, we describe a protocol for the establishment of a standardized national disease-specific dataset for patients with SLE—the Systemic Erythematous Lupus Network (LUNET) Registry—which will facilitate data sharing, cross-comparison, and interoperability among centers. The LUNET project is designed to provide high-quality, robust data to enhance our comprehension of SLE, evaluate the short and long-term safety and effectiveness of various treatment approaches, and globally improve SLE patient management in the real-life setting.

The primary objective of this initiative is to establish a national disease-specific dataset, designed to collect comprehensive clinical information from the largest feasible cohort of patients with SLE. This resource will support robust statistical analyses, and promote the integration of evidence-based findings into routine clinical practice. We primarily aim to gather real-world data from as many patients as possible in order to describe the different clinical presentations of SLE in the Italian population. This will expand the current knowledge regarding SLE clinical variability and possible presentations in a manner that will be more efficient and provide more detailed information, compared to traditional clinical research, which often relies on single-center data. Our specific objectives are as follows:

- Planning and conducting epidemiological studies to evaluate the incidence and prevalence rates of different clinical manifestations of SLE, and to evaluate short- and long-term outcomes, including survival rates and organ dysfunction, injury, and failure
- Promoting multicenter studies that will compare different patient subgroups based on clinical phenotypes and organ involvement, to attain insights regarding disease expression and progression
- Describing the profile of patients with lupus nephritis in the national population, to improve guidelines on its systematic and standardized assessment.
- Evaluating the presence of central and peripheral nervous system involvement in the national population of patients with SLE, assessing the type, frequency, and severity of neurological symptoms and objective signs, as well as the instrumental techniques used in clinical practice (e.g., Magnetic Resonance Imaging (MRI)
- Assessing biomarker data to support early diagnosis and guide treatment choices
- Improving screening techniques for early identification of organ damage in SLE patients
- Improving early treatment strategies
- Tracking adherence and response to therapy
- Evaluating the short- and long-term real-life effectiveness and safety of old and novel drugs approved for SLE, overall and in groups of patients with specific phenotypes
- Assessing SLE patients’ quality of life according to clinical phenotype and treatment approach
- Assessing how socioeconomic status impacts patients’ access to healthcare and absenteeism in relation to the disease and various therapeutic approaches
- Evaluating how different therapeutic strategies impact hospitalization
- Assessing the influence of old and novel therapies on fertility, pregnancy, and breastfeeding
- Monitoring cardiovascular risk in SLE patients by evaluating the associated comorbidities, prospective development of cardiovascular events, and biomarker levels (e.g., fasting blood glucose, hemoglobin A1c, total cholesterol, HDL cholesterol, LDL cholesterol, triglycerides and pro-BNP)
- Evaluating bone metabolism in patients with SLE through assessment of laboratory measurements (e.g., vitamin D, calcium, phosphorus, calciuria, and phosphaturia) and instrumental assessments, such as dual-energy X-ray absorptiometry (DEXA) scans
- Monitoring the causes of death among SLE patients

Here, we describe the LUNET protocol, which was designed to characterize and manage the heterogeneity of SLE pathology. The primary outcome was defined as the incidence and prevalence rates of various clinical manifestations of SLE. The clinical spectrum of SLE in the national population will be comprehensively assessed, including both frequent and rare manifestations, in recognition of the disease’s multisystemic nature. Particular focus will be placed on lupus nephritis and neuropsychiatric involvement, given their role as key indicators of disease severity and their impact on prognosis. Given this largely descriptive primary objective, we will evaluate the adequacy of the sample size over the course of the study, with consideration of both the quantity and quality of the collected data.

## 2. Materials and Methods

### 2.1. Registry Design and Governance

The LUNET registry will adopt a multicenter observational prospective methodology designed to create a network of reference centers with specific expertise in managing SLE patients, with the aim of compiling the most comprehensive possible data on the clinical, laboratory, and instrument-measured characteristics of this disease in a real-world setting. To meet this objective, an extensive database of patients with SLE, according to SLICC 2012 [1] and/or ACR/EULAR 2019 [2], will be established and analyzed to gain insight into various clinically and scientifically relevant aspects of SLE.

This study is promoted by the Italian Society of Allergy, Asthma, and Clinical Immunology (SIAAIC), which was founded in 1953, and is the largest Italian scientific society dedicated to allergy and clinical immunology. The SIAAIC has about 1800 active members affiliated with universities, hospital departments, and territorial outpatient clinics. Its main aim is to advance allergy and clinical immunology as a discipline via integrated efforts in specialist and post-specialist education, promotion of knowledge in this field, the conduct of clinical and epidemiological research, and support of patient organizations (https://siaaic.org/; accessed on 13 March 2025).

### 2.2. Oversight

**Figure 1:**
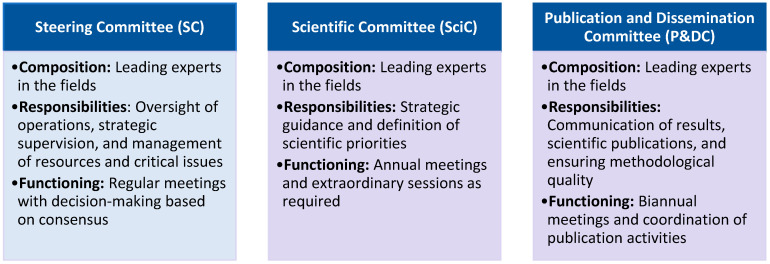
The three committees responsible for governance of the LUNET registry.

The LUNET Study is overseen by three governing bodies: the LUNET Steering Committee (SC), the LUNET Scientific Committee (SciC), and the Publication and Dissemination Committee (P&DC) (Figure 1).

**Figure 2:**
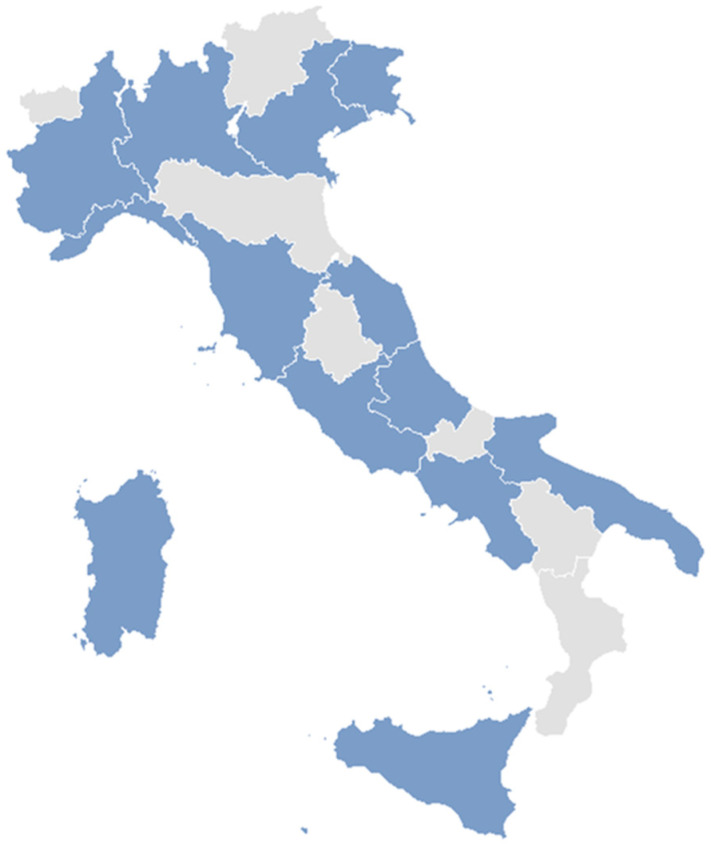
Geographic distribution of referral centers currently involved in the LUNET project.

The LUNET Network currently comprises 18 centers across Italy, and involves leading experts in clinical immunology, internal medicine, and rheumatology (Figure 2).

The LUNET Network remains open to the inclusion of additional centers to continuously expand the dataset and improve the representativeness of its real-world clinical evidence. The primary requirement for participating centers is a specific expertise in diagnosing and managing SLE patients. Specific selection criteria include: access to an active cohort of patients with SLE sufficient to ensure regular enrollment and follow-up; availability of qualified personnel such as clinical investigators and research nurses; a dedicated outpatient clinic for systemic autoimmune diseases; access to biotechnological and investigational drugs; Day Hospital/Day Service activities; ward support for standard inpatient admissions and/or dedicated beds; and availability of instrumental diagnostics (e.g., capillaroscopy, spirometry, ultrasound, renal biopsy, etc.).

### 2.3. Patients

Patients will be enrolled from the Italian Referral Center of Clinical Immunology, Rheumatology, and Internal Medicine, which has specific expertise in SLE management. The registry will enroll consecutive patients, who must meet all of the following inclusion criteria: age ≥ 18 years; confirmed SLE diagnosis according to the SLICC 2012 [1] and/or ACR/EULAR 2019 classification criteria [2]; and provision of signed written informed consent prior to participation. Written informed consent will be obtained after participants are given a comprehensive explanation of the registry’s objectives, emphasizing that participation will not influence clinical management or treatment. Participants will be informed of their right to withdraw consent at any time without repercussion, and will be assured that all data will be handled in compliance with applicable laws, to ensure privacy, pseudonymization, and data security. Once the inclusion criteria are fulfilled, no exclusion criteria will preclude the patients’ enrollment.

### 2.4. Database

**Figure 3:**
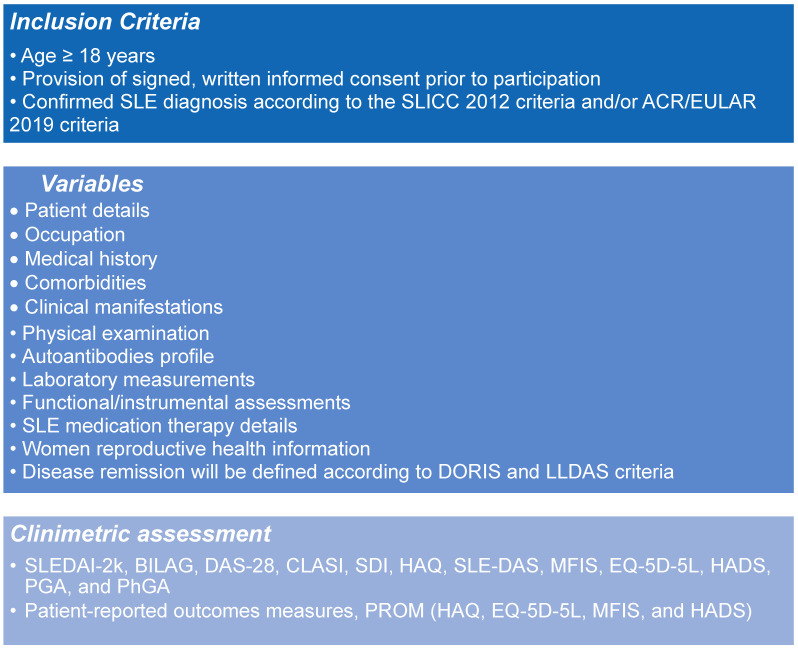
List of all systemic lupus erythematous (SLE) variables included in the LUNET registry.

Patients’ data will be collected at enrolment and every 12 months thereafter, for a maximum follow-up period of 120 months (10 years). Patient data will be collected in a pseudonymized form, ensuring that individuals cannot be directly identified. The dataset will include the following information (Figure 3):

- Inclusion criteria
- Patient details
- Occupation
- Medical history
- Comorbidities
- Clinical manifestations, including symptoms and signs of organ involvement at disease onset and subsequent follow-up, course of the disease, and number of disease flares per year
- Physical examination and clinimetric assessment results (SLEDAI-2k, BILAG, DAS-28, CLASI, SDI, HAQ, SLEDAS, MFIS, EQ-5D-5L, HADS, PGA, and PhGA) [12,13,14,15,16,17,18,19,20]
- Autoantibodies profile, including positivity of ANA, anti-dsDNA, anti-Sm, anti-Ro/SSA, anti-La/SSB, anti-centromere, anti-Scl70, anti-RNA polymerase III, anti-RNA polymerase I, anti-U3 RNP/fibrillarin, Jo1, anti-histone, anti-CCP, rheumatoid factor, ANCA-MPO, ANCA-PR3, myositis antibodies (i.e., anti-Mi2, anti-PL7, anti-PL12, anti-OJ, EJ, SRP, anti-MDA5, Anti-NXP2, and TIF1-gamma), and other anti-nuclear antibodies.
- Laboratory measurements: white blood cells (cells/mm^3^), red blood cells (cells/mm^3^), hemoglobin (g/dL), platelets (cells/mm^3^), lymphocytes (cells/mm^3^), neutrophils (cells/mm^3^), C-reactive protein (mg/dL), erythrocyte sedimentation rate value (mm/h), C3 (mg/dL), C4 (mg/dL), creatine kinase (mg/dL), NT-proBNP (pg/mL), BNP (pg/mL), total cholesterol (mg/dL), low density lipoprotein (mg/dL), high density lipoprotein (mg/dL), triglycerides (mg/dL), fasting blood glucose (mg/dL), glycated hemoglobin (mmol/mol, %), AST (U/L), ALT (U/L), GGT (U/L), eGFR (mL/min/1.73 mq), azotemia (mg/dL), serum creatinine (mg/dL), urine protein creatinine ratio (mg/g), urine sediment analysis (physical, chemical and microscopic analysis of urine), 24 h urine protein test (mg/day), vitamin D (mg/dL), calcium (mg/dL), phosphorus (mg/dL), calciuria (mg/24 h specimen), and phosphaturia (mg/24 h specimen)
- Functional/instrumental assessments: electrocardiography, echocardiography, kidney biopsy, musculoskeletal ultrasound and MRI, brain MRI, cerebrospinal fluid analysis, dual-energy X-ray absorptiometry (DEXA) scans
- SLE medication therapy details, including category, therapy name, current, start date, stop date, precise dose, dose unit, drug side effects, reason for change in therapy, and others
- Women’s reproductive health information, including the number of pregnancies, miscarriages, live births, congenital abnormalities, complications during pregnancy, administration of drugs before and during pregnancy, breastfeeding duration, administration of drugs before and during breastfeeding, and use of any contraceptive measures
- Disease remission will be defined according to the Definition of Remission in Systemic Lupus Erythematosus (DORIS) criteria [20] and Lupus Low Disease Activity State (LLDAS) [21]

### 2.5. Pharmacovigilance

Data will be collected and recorded to monitor the frequency and grading of immediate and delayed, local and systemic adverse reactions attributable to the administration of any drugs, as well as the resulting therapeutic management of such events (https://www.aifa.gov.it/content/segnalazioni-reazioni-avverse; accessed on 13 March 2025).

### 2.6. Statistical Analysis

Sample size and power calculation will be defined according to the primary outcome pertaining to each specific registry study conducted on the LUNET registry. Additionally, statistical analyses will be performed in accordance with the specific nature and type of data under investigation, and the specific objectives of the studies conducted on behalf of the LUNET Network. Descriptive analyses will be performed to determine the main characteristics of patients, including sex, age, comorbidities, disease severity, outcomes, ongoing therapies, etc. Continuous variables will be expressed as mean ± standard deviation, or median and interquartile range if asymmetrically distributed. Categorical variables will be reported as absolute frequencies and percentages. The assumption of normality will be checked using the Shapiro–Wilk test. If normality is verified, parametric tests will be used. Correlations and associations between the investigated socio-demographic factors and clinical end-points (expressed as continuous, categorical, or time-to-event variables) will be assessed using appropriate statistical methods, including but not limited to crude and adjusted regression models. Details regarding the statistical analyses performed will be provided in future papers. The level of significance will be set at α = 0.05. Data collected in the registry will be managed by statisticians and physicians involved in the network.

### 2.7. Electronic Database, Data Management, and Confidentiality

All data will be collected and stored using an electronic case report form (eCRF) implemented by Research Electronic Data Capture (REDCap^®^; v15.0.21), as installed by the SIAAIC. REDCap^®^ is a secure web-based software platform, designed in 2004 at Vanderbilt University Medical Center, for constructing and managing online surveys and databases. REDCap platform use is free to all members of the REDCap consortium. Established in 2009, the REDCap consortium currently includes over 7700 institutions from 160 Countries (available at https://projectredcap.org/; accessed on 22 March 2025). Notably, the software has evolved into a neutral data collection platform that can capture and support any type of data collection (available at https://projectredcap.org/; accessed on 22 March 2025). Notably, REDCap^®^ provides the following features: an intuitive user interface for data collection; audit trails to record any data manipulation and data export procedures; automatic export procedures in pseudonymous or anonymous format compatible with the most common statistical software; and data integration procedures and interoperability with external resources [22,23].

Each user affiliated with a LUNET Center will receive a unique username and will set a personal password. This “table-based” authentication system will ensure that passwords are securely stored in the database using the SHA-512 hash function. Authentication is further secured by an HTTPS connection secured with an SSL certificate. Users can enter their credentials to access a dedicated area of the web application.

The permissions granted to each user depend on their role assigned by the administrator (data entry, data manager, principal investigator, etc.). The application generates a unique identification code for each patient. Each center must locally maintain the enrollment list, with patient identification data associated with a unique code (pseudonymization). No patient identification data will be stored in the database, in accordance with the principle of data minimization.

Data privacy is strictly maintained. Investigators at each center—including principal and site investigators—will only have access to the data from their own center, and will not be able to view data collected at other centers. All data will be processed in accordance with the General Data Protection Regulation (GDPR; EU Regulation 2016/679), Legislative Decree no. 101 of 10 August 2018, and all other applicable national regulations and subsequent amendments.

To ensure the reliability and consistency of data collected across all participating centers, the registry will implement a structured quality management approach. Data will be collected using standardized eCRFs within REDCap^®^, supported by a detailed user manual to ensure uniform data entry and reduce variability due to differences in clinical documentation practices. Although laboratory tests are performed locally as part of routine clinical care, several measures will harmonize and contextualize laboratory data, including: use of standardized units and reference ranges, with conversion rules applied centrally when needed; collection of relevant metadata for each analyte, such as the analytical method (e.g., ELISA), local laboratory reference ranges, test date, sample origin, and center-specific information; implementation of automated plausibility checks to flag biologically implausible or out-of-range laboratory values; and internal consistency checks across multiple timepoints for individual patients to identify inconsistencies or potential data entry errors. Regular data reviews and central monitoring will be conducted to ensure data completeness, accuracy, and overall quality. These procedures aim to enable robust use of real-world laboratory data, even without centralized laboratory testing, while maintaining high data integrity and analytical reliability.

### 2.8. Ethics

The study will be conducted in accordance with the principles and procedures of good clinical practice (GCP) and the latest revision of the Declaration of Helsinki. It has also been designed to comply with applicable Italian regulations—including the ICH Harmonized Tripartite Guidelines for Good Clinical Practice (1996); Directive 91/507/EEC; The Rules Governing Medicinal Products in the European Community; Legislative Decree No. 211 of 24 June 2003; Legislative Decree No. 200 of 6 November 2007; the Ministerial Decree of 21 December 2007; the General Data Protection Regulation (GDPR); and Legislative Decree No. 101 of 10 August 2018.

This is a non-interventional study. Demographic, clinical, and therapeutic data will be collected as part of routine diagnostic, clinical, and therapeutic practices that are typically carried out in the real-life management of patients with SLE, with the aim of providing optimal patient care. The therapeutic approaches adopted for enrolled patients will not be influenced by study participation and will remain entirely at the discretion of the treating physician, based on current clinical guidelines and the individual clinical context.

The investigator will be responsible for obtaining informed consent and privacy authorization from each patient prior to data collection, and for making every reasonable effort to ensure that the patient fully understands the provided information. The patient’s signature will confirm their understanding of the study information and consent contents. The investigator must also sign and date the consent form. The original signed document will be retained by the investigator, and a copy will be provided to the patient.

The study protocol has been approved by the Ethics Committee of the University of Naples Federico II (protocol code No. 30/2025, dated 2 April 2025). Enrollment at other centers will commence following approval by each respective local Ethics Committee.

## 3. Discussion

National disease-specific datasets and registries are crucial for clinical research, since they can provide real-world and long-term data about clinical aspects, biomarkers, and treatments. Registries collect data from actual patients over time, outside the controlled environment of randomized controlled trials (RCTs). This can help enhance our understanding of the natural history of a disease, can provide information about how treatments work in everyday settings, and can elucidate potential variations in care and outcomes across different geographic areas. Indeed, while RCTs often run for limited time periods, registries can follow patients for many years and can thus help assess the long-term safety and effectiveness of treatments, the prevalence of disease recurrence or progression, and quality of life over time. Moreover, RCTs often involve rigid inclusion and exclusion criteria, leading to the exclusion of groups of patients with a higher risk of infection, with comorbidities, or showing particular clinical phenotypes of a disease. For example, patients with NPSLE have been excluded from belimumab and anifrolumab RCTs, such that our knowledge regarding the use of these drugs in this patient subset remains limited to case reports and case series. Additionally, the therapeutic protocols used in RCTs do not always enable evaluation of the long-term effects of a drug that may be observed in routine clinical practice. This is particularly relevant when considering that patients with a higher probability of receiving new treatments are often those with greater resistance to other treatments and a higher risk of adverse events. The occurrence of rare or late events can only be adequately assessed through large observational studies with long follow-up periods. From this perspective, registries support post-marketing surveillance by monitoring drug performance in the general population after approval. Finally, disease-specific registries can also guide future clinical trials, facilitating better-targeted design and faster recruitment, as they can help with the identification and characterization of patient groups having specific conditions.

Over time, other research groups have tried to design disease-specific registries for SLA, often with a focus on limited aspects of SLE or specific outcomes. For example, the British Isles Lupus Assessment Group Biologics Register (BILAG-BR) is a UK-based prospective registry established to monitor and evaluate the safety and effectiveness of biologic therapies in patients with SLE [24]. The Lupus Erythematodes Langzeit-Studie (the LuLa cohort) is a German study that primarily collects patient-reported outcomes to assess various aspects of living with SLE—including disease burden, therapy adherence, coping mechanisms, and quality of life [25,26,27,28]. The Swiss Systemic Lupus Erythematosus Cohort Study (SSCS) is a nationwide multidisciplinary research initiative with the aim of enhancing the understanding of SLE in Switzerland. Launched in 2007, the SSCS involves multiple Swiss medical centers specializing in immunology, nephrology, internal medicine, and rheumatology, and is coordinated by the Association of the Swiss SLE Cohort Study (ASSCS) [29]. Another prominent national SLE registry from a European country is the lupus register of the Spanish Society of Rheumatology (RELESSER) [30], which is one of the largest and most comprehensive SLE registries. The RELESSER includes data from over 4000 patients across 45 centers, and records 359 variables per patient, including demographics, clinical manifestations, disease activity, treatments, and outcomes. RELESSER has substantially contributed to our understanding of SLE in Spain, providing valuable insights into several aspect of the disease, such as pregnancy [31], cardiovascular events [32], infections [33], diffuse alveolar hemorrhage [34], early- and late-onset disease features [35], and gender-related phenotypic and laboratory manifestations of this disease [36].

Another notable model is the Lupus Family Registry and Repository (LFRR) in the United States [37]. Formerly known as the Lupus Multiplex Registry and Repository (LMRR), the LFRR was a research initiative established in 1995, which focused on advancing the understanding of SLE through the collection and analysis of clinical and biological data [37]. The LFRR aimed to investigate the genetic underpinnings of SLE by collecting and distributing data and materials from families with one or more living members diagnosed with the disease [37]. Additionally, the Lupus Foundation of America (LFA) has supported various research initiatives—including the COVID-19 Global Rheumatology Alliance Registry, which collects data on patients with rheumatic diseases, including lupus, who have been diagnosed with COVID-19 [38]. This registry provides insights into how the pandemic has affected individuals with autoimmune conditions [38]. One registry with a special focus on treatments is the International Registry for Biologics in Systemic Lupus Erythematosus (IRBIS), which is a global multicenter initiative established by the Systemic Lupus International Collaborating Clinics (SLICC) group in 2010 [39]. The primary aim of this ongoing effort is to systematically collect and analyze data regarding the use of biologic therapies in patients with SLE, particularly those undergoing off-label treatment, such as with rituximab, abatacept, etanercept, and adalimumab.

The first attempt to establish an Italian national registry was the Lupus Italian REgistry (LIRE), initiated by the Italian Society for Rheumatology (SIR) [40]. LIRE was a multicenter prospective registry that was active from 2015 to 2021, with the aim of evaluating therapeutic strategies used in daily clinical practice, and their relationships with disease activity and outcomes. Since publication of the LIRE results, the therapeutic armamentarium available for SLE patients has significantly widened [5], with the approval of novel drugs, such as anifrolumab [41,42] and voclosporin [43,44]. Additionally, belimumab has been approved for SLE-related renal involvement [5], and several other molecules are under investigation, highlighting the need for large networks designed to collect real-life data. Another multicenter study, the Early Lupus Project, included nine Italian centers and focused on patients with recent-onset SLE, with the aim of gathering information about the main clinical and serological characteristics at the beginning of the disease, to obtain detailed data regarding early disease manifestations [45]. Lastly, another Italian research group utilized primary care databases to explore the epidemiology of SLE in Italy, and to attain insights into the demographic and clinical characteristics of newly diagnosed SLE patients during the period 2017–2022 [46]. Their research utilized the Health Improvement Network (THIN^®^) database, which is a large standardized European network of fully anonymized longitudinal primary care data [46]. However, that study relied only on general practitioner data, rather than using linked primary and secondary care data. As discussed by the authors, this approach could lead to underestimation of SLE incidence and prevalence rates, especially because some elements of the EULAR/ACR classification criteria may not be recorded in the Italian THIN database [46]. Therefore, the authors concluded that their estimates could be refined by further epidemiological studies, including larger population samples.

## 4. Conclusions

National and international disease registries and networks have played pivotal roles in advancing our understanding of heterogeneous diseases, such as SLE. The LUNET project is intended to serve as an active primary source of real-world data about SLE patients across Italy and to support clinical research by providing long-term insights and capturing diverse patient experiences that are often underrepresented in traditional clinical trials. Unlike initiatives focused on specific disease aspects, the LUNET registry is designed to collect comprehensive data about clinical manifestations, therapeutic interventions, and outcomes, thereby facilitating the development of personalized treatment strategies grounded in real-world evidence. Furthermore, the broad geographic coverage of the registry will help standardize data collection across regions, reduce variability, minimize bias, and ensure sufficient statistical power to address a wide range of clinical and research questions. The LUNET registry is not only comprehensive but also innovative in its scope and real-time data collection, which allows for more accurate monitoring of disease progression and treatment effectiveness in everyday clinical practice. This represents a crucial step forward in bridging the gap between clinical trials and real-world patient management. For clinicians, the registry provides actionable insights that can inform tailored treatment decisions, optimize patient outcomes, and enhance the overall quality of care. By capturing longitudinal data across diverse patient populations and healthcare settings, LUNET empowers healthcare providers with evidence-based tools to better anticipate disease flares, manage comorbidities, and personalize therapeutic approaches in SLE. Ultimately, the establishment of this national network will foster international collaboration, enabling the identification of shared and divergent disease patterns and contributing to improved global management of this highly heterogeneous condition.

## Data Availability

The original contributions presented in this study are included in the article. Further inquiries can be directed to the corresponding author.
